# Population-Based Studies on the Epidemiology of Insulin Resistance in Children

**DOI:** 10.1155/2015/362375

**Published:** 2015-07-27

**Authors:** M. P. van der Aa, S. Fazeli Farsani, C. A. J. Knibbe, A. de Boer, M. M. J. van der Vorst

**Affiliations:** ^1^Department of Pediatrics, St. Antonius Hospital, P.O. Box 2500, 3430 EM Nieuwegein, Netherlands; ^2^Division of Pharmacoepidemiology and Clinical Pharmacology, Utrecht Institute for Pharmaceutical Sciences (UIPS), Utrecht University, P.O. Box 80082, 3508 TB Utrecht, Netherlands; ^3^Department of Clinical Pharmacy, St. Antonius Hospital, P.O. Box 2500, 3430 EM Nieuwegein, Netherlands

## Abstract

*Background*. In view of the alarming incidence of obesity in children, insight into the epidemiology of the prediabetic state insulin resistance (IR) seems important. Therefore, the aim of this systematic review was to give an overview of all population-based studies reporting on the prevalence and incidence rates of IR in childhood. *Methods*. PubMed, Embase, and Cochrane library were searched in order to find all available population-based studies describing the epidemiology of IR in pediatric populations. Prevalence rates together with methods and cut-off values used to determine IR were extracted and summarized with weight and sex specific prevalence rates of IR if available. *Results*. Eighteen population-based studies were identified, describing prevalence rates varying between 3.1 and 44%, partly explained by different definitions for IR. Overweight and obese children had higher prevalence rates than normal weight children. In seven out of thirteen studies reporting sex specific results, girls seemed to be more affected than boys. *Conclusion*. Prevalence rates of IR reported in children vary widely which is partly due to the variety of definitions used. Overweight and obese children had higher prevalence and girls were more insulin resistant than boys. Consensus on the definition for IR in children is needed to allow for comparisons between different studies.

## 1. Introduction

Nowadays, the body mass index (BMI) is increasing in many populations and childhood obesity is an emerging problem [1–3]. In the United States the prevalence rates of obesity between 1971 and 1974 in 6–11-year-old white/black children were 4%. Between 1999 and 2002, these prevalence rates increased to 13% and 20% in white and black children, respectively [4]. In 2012 the overall prevalence rate of obesity in 2–19-year-old American children was 17.3% [1]. In developing countries the prevalence rate of overweight and obesity in preschool children (<5 years old) in 2010 was estimated to be 6.1% and 11.7%, respectively [5]. Moreover, the prevalence of overweight in children <5 years of age raised in the African continent between 2000 and 2013 from 5.1 to 6.2% (+1.1%), while in the American Continents, the prevalence increased with 0.5% (6.9 to 7.4%). (http://apps.who.int/gho/data/view.main.NUTWHOOVERWEIGHTv?lang=en).

The rising prevalence of obesity will cause an increase in obesity related complications such as insulin resistance (IR), hypertension, dyslipidemia, and type 2 diabetes mellitus (T2DM) [6, 7]. The energy excess in obesity may result in hyperplasia and hypertrophy of adipocytes, leading to oxidative stress. This oxidative stress of adipocytes induces a chronic low-level inflammation in adipose tissue and production of adipokines, free fatty acids, and inflammatory mediators. This inflammation is related to peripheral IR, IR of hepatocytes, and impaired insulin secretion by the pancreatic beta cells. Finally, this process causes dysregulation of glucose homeostasis and development of T2DM [8]. Although obesity plays a key role in the pathophysiology of IR, IR is an independent risk factor for cardiovascular and metabolic diseases [9–12]. Therefore, it is important to know the extent of IR in pediatric populations. Knowledge on the prevalence rates of IR and its clinical consequences during childhood will increase the awareness of physicians and other health care professionals. Despite the reported association between IR and increased cardiovascular risk in pediatric populations [13], there is no overview of data on the epidemiology of IR in this population. Many studies focus on the extent of IR in overweight and obese populations, but limited studies have a population-based study design.

The aim of this study is to systematically review all available population-based studies on the epidemiology of IR in pediatric populations. We will describe the weight and sex specific prevalence and incidence rates of IR in the included studies, together with the study-specific definition used to define IR.

## 2. Methods

### 2.1. Systematic Search and Study Selection

This review follows the guidelines of “Meta-analysis of Observational Studies in Epidemiology” (MOOSE) [32]. A systematic search was conducted in PubMed, Embase, and the Cochrane library, using the search strategies as displayed in Table 1. The search was performed in December 2014 and covered all publications in the time period between the inception of each database and the search date. All articles in English, French, German, Spanish, and Dutch languages were included and their title and abstract were screened to find the relevant studies. All results were imported into a RefWorks file (http://www.refworks.com/) and duplicate articles were removed. Subsequently, the title and abstract of all unique results were screened using the exclusion criteria. Articles were excluded if they were review articles, studied a population older than 19 years, or did not report prevalence and/or incidence rates of IR in the abstract. Furthermore, all conference abstracts without a full text publication were excluded. All available full text articles were retrieved and their design was scrutinized to select population-based studies. The reference lists of all included population-based studies were investigated to find relevant articles not included in the original search.

### 2.2. Data Extraction and Analysis

Data were extracted on the study design, sample size, calendar time of data collection, mean age of participants, ethnicity, criteria used to determine IR (method and cut-off value), prevalence, and incidence rates of IR in the complete study population, and if available in subpopulations based on weight category (normal weight, overweight, and obesity), and sex. Data were entered in an excel file. Pooling of data was not possible because of the large variability in study design, population, and definitions used to determine IR. Data are presented in a descriptive manner.

## 3. Results

### 3.1. Systematic Search and Study Selection

**Figure 1:**
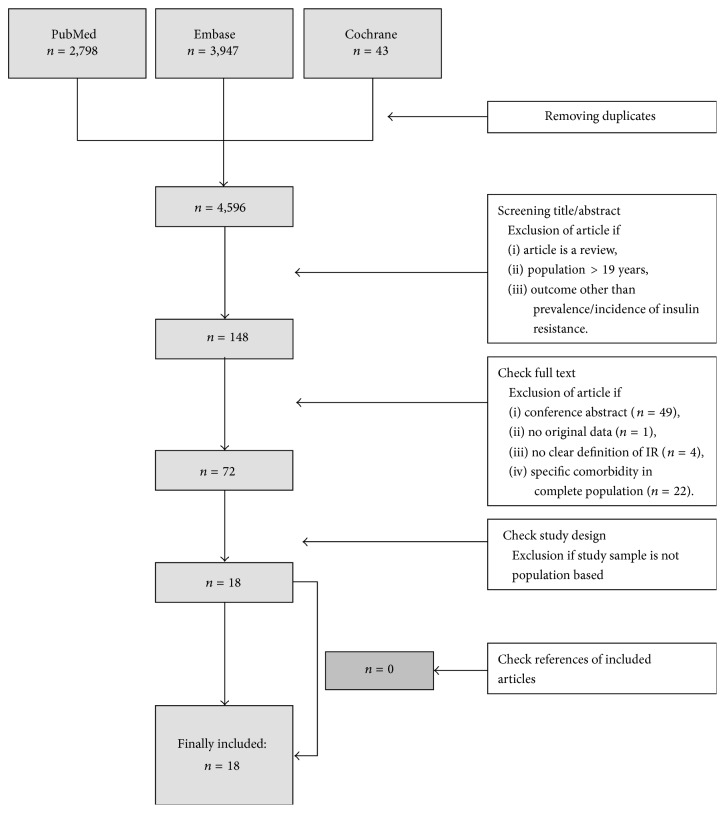
Flowchart of search and included studies.

With the search strategy presented in Table 1, in PubMed, Embase, and Cochrane 6,788 articles (with 4,596 unique studies) were retrieved. Screening of titles and abstracts led to the exclusion of 4,448 articles (Figure 1). The full text of the 148 remaining articles was checked and 76 articles were excluded based on our exclusion criteria. Critical appraisal of the 72 remaining articles resulted in the final inclusion of 18 population-based studies. All included studies reported prevalence rates of IR and none of them reported incidence rates. An overview of the included studies and extracted data is presented in Supplemental Table 1 (see Supplemental Table 1 in the Supplementary Material available online at http://dx.doi.org/10.1155/2015/362375).

### 3.2. Study Characteristics

The 18 included studies were performed in 13 countries. Except for the African continent, all continents are represented. The studies were performed between 1999 and 2011. Sample sizes varied from 80 to 3,373 children [14, 28]. Most studies recruited their study population at selected schools [14–25, 29–31]. The New Zealand study population were volunteer adolescents who were recruited by Pacific Island community workers, even though it was not reported where they recruited the participants [28].

In the majority of the studies (*n* = 14), the age of the study participants was above 10 years [15, 17–21, 23–26, 28–31]. Four studies included also children younger than 10 years, with ranges that varied between 6 and 19 years [14, 16, 22, 27]. Ethnicity was not reported in 50% of the studies. All study characteristics are presented in Supplemental Table 1.

### 3.3. Methods and Cut-Off Values to Define IR

In the studies, six different methods were used to determine IR (Table 2). These methods were Homeostasis Model Assessment Insulin Resistance (HOMA-IR), fasted plasma insulin (FPI), Quantitative Insulin Sensitivity Check Index (QUICKI), fasted glucose/insulin ratio (FGIR), HOMA2, and the McAuley index. All these indices are based on FPI; for HOMA-IR, QUICKI, FGIR, and HOMA2 fasted plasma glucose (FPG) values are also needed (Table 2). The McAuley index is the only index for which fasted triglycerides are required besides FPG and FPI. None of the above-mentioned equations use anthropometric measurements or values derived from an oral glucose tolerance test.

HOMA-IR, FPI, and QUICKI were the most frequently used methods to determine IR (HOMA-IR: *n* = 14 [14–27]; FPI: *n* = 7 [17, 19, 20, 28–31]; QUICKI *n* = 2 [17, 23], Table 2).

The cut-off values used to define IR for HOMA-IR ranged from 2.1 to 5.56, while for FPI cut-off values varied between 9.85 and 23.7 *μ*U/mL (corresponding with 68.4 and 164.8 pmol/L, resp.) (Table 2). The study of Budak et al. used a cut-off value different from the other studies, as their definition for IR was a HOMA-IR <3.16 which was in contrast with other studies that defined IR as HOMA-IR greater than a specific value [18]. We did not succeed to contact Budak et al. to verify this cut-off value.

Age and sex specific cut-off values were reported in, respectively, one [30] and three studies [20, 24, 30]. Girls had higher cut-off values for FPI and HOMA-IR compared with boys. For both sexes, adolescents aged 14-15 years had the highest cut-off values for FPI [30].

### 3.4. Prevalence of IR

**Figure 2:**
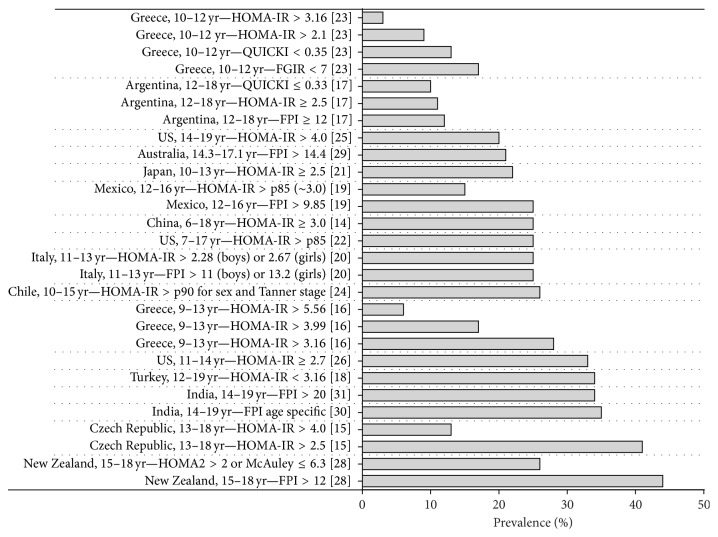
The overall prevalence rates (%) of IR in the included studies.

The overall prevalence rates of IR in 17 out of 18 population-based studies are presented in Figure 2. The study of Ranjani et al. only reported sex specific prevalence rates [27]. The lowest prevalence rate of IR was reported from Greece with 3.1% in children aged 10–12 years (using the cut-off value of HOMA-IR > 3.16 for IR, Figure 2) [23]. In the same study population, three other definitions of IR (HOMA-IR > 2.1, QUICKI < 0.35, and FGIR < 7) were applied resulting in prevalence rates of 9.2, 12.8, and 17.4%, respectively.

The highest prevalence rate of IR was reported by Grant et al. for the 15–18-year-old Pacific Island adolescents in New Zealand [28]. They reported a prevalence rate of 44% with IR defined as FPI > 12 *μ*U/mL. This definition of IR has been used in another study by Bonneau et al. which resulted in a prevalence rate of 11.7% for the 12–18-year-old Argentinian adolescents [17].

### 3.5. Sex and Weight Specific Prevalence of IR

**Figure 3:**
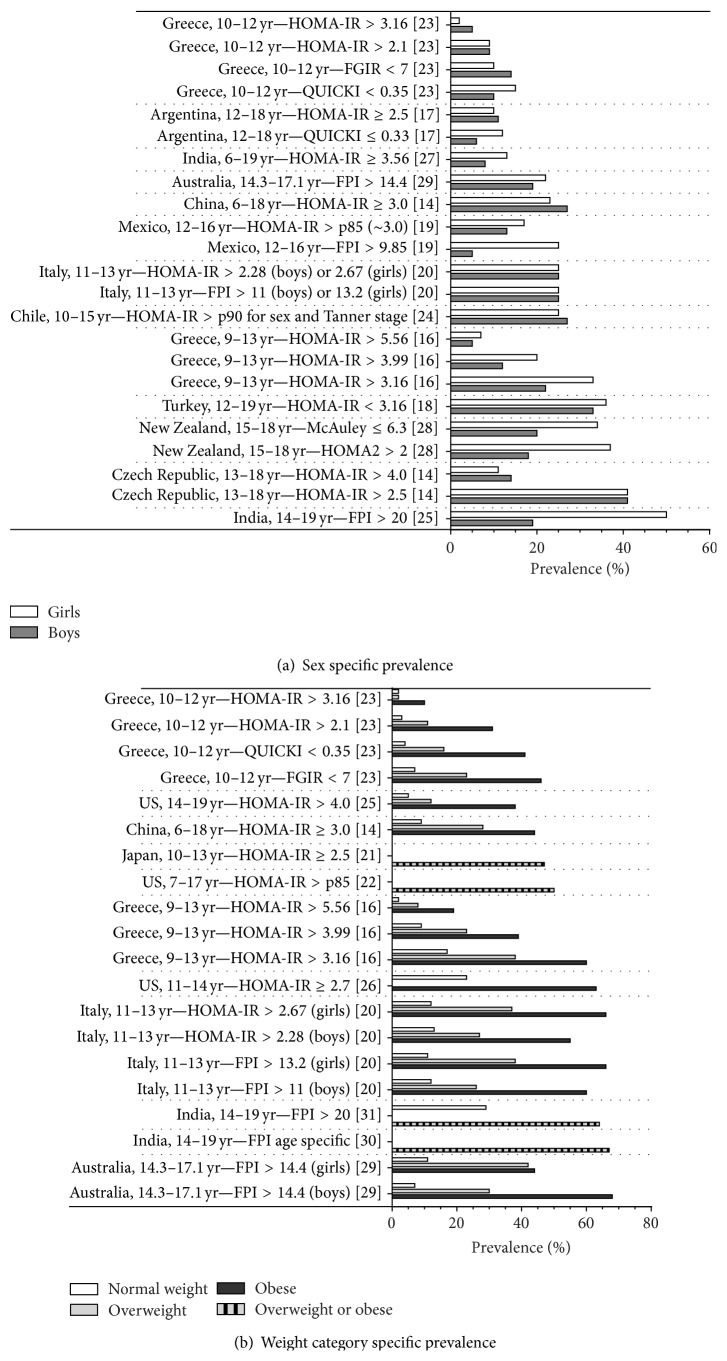
Prevalence of IR by sex (a) and weight category (b).

Thirteen studies reported separate prevalence rates for boys and girls (Figure 3(a)). In 7 out of 13 studies, IR was more prevalent in girls [16, 18, 19, 27–29, 31]. Three studies reported higher prevalence rates for boys [14, 15, 17]. In one study the prevalence rate of IR was similar for boys and girls [20]. In two studies it depended on the criteria used to determine IR whether boys or girls were having the highest prevalence rates [17, 23].

Figure 3(b) shows the influence of weight (normal, overweight, and obesity) on the prevalence of IR. A major difference was observed between normal weight and obese populations. Normal weight populations had substantial lower prevalence rates of IR, irrespective of the used definition for IR. The maximum difference in weight specific prevalence rates of 61.3% was reported in Australian boys, with prevalence rates in normal weight and obese boys of 7.1% and 68.4%, respectively [29].

## 4. Discussion

To the best of our knowledge, this is the first systematic review summarizing all available population-based studies on the epidemiology of IR during childhood. While we could not find any population-based study reporting the incidence rate of IR in children, the reported prevalence rates varied between 3.1% in Greek children and 44% in Pacific Island teenagers living in New Zealand. There was not only variation in the prevalence rates of IR, but we also observed that these 18 included studies used 6 different methods combined with diverse cut-off values to determine IR. For instance, the FPI cut-off values varied between 9.85 and 23.7 *μ*U/mL (corresponding with 68.4 and 164.8 pmol/L, resp.) [19, 30] and the HOMA-IR cut-off values ranged between 2.1 and 5.56 [16, 23]. The lack of a uniform definition and cut-off value to determine IR impedes pooling of data, therefore reporting on overall prevalence rates.

Although substantial variation in the prevalence rates of IR could be partly explained by differences in the study population characteristics (e.g., age, weight, ethnicity, pubertal status, etc.), the use of different methods and cut-off values to determine IR may play an important role as well. As an example, in the study by Manios et al. in 481 Greek school children, different methods resulted in various prevalence rates (i.e., 3.1 versus 12.8 and 17.4% for HOMA-IR, QUICKI, and FGIR, resp., Figure 2) [23]. Even if studies use the same method to measure IR, different cut-off values impede comparison between studies. Again, in the study by Manios et al., the use of different cut-off values for HOMA-IR method (>3.16 and >2.1) in the same study population resulted in prevalence rates of 3.1 and 9.2%, respectively [23]. A lower cut-off value results in a higher prevalence rate of IR and vice versa.

The highest reported prevalence rate for IR was 44% in Pacific Island teenagers (New Zealand) [28]. In that study IR was defined as FPI > 12 *μ*U/mL, which is a relatively low cut-off value that might contribute to the high reported prevalence rate. In another study in Mexico, which used the lowest cut-off value for FPI (FPI > 9.85 mU/L) a prevalence rate of 24.8% was reported [19]. When the same cut-off values would have been used in these two studies, the difference in prevalence rates would even have been larger. Even though the difference between these two populations cannot be quantified precisely, not only because of different cut-off values, but also because other factors such as age, weight, and pubertal stage were not taken into account, this analysis shows that prevalence rates of IR are variable in different populations, which was also observed in other studies.

Overweight or obesity is an important factor influencing the prevalence of IR. The effect of overweight or obesity on IR is clearly observed in all presented studies as prevalence rates in overweight or obese children and adolescents were reported to be higher than in normal weight children and adolescents (Figure 3(b)). Most studies (7 out 11 studies presenting weight specific prevalence rates) not only differentiated between normal weight and overweight/obesity, but also stratified into normal weight, overweight, and obese children and adolescents [14, 16, 20, 23, 25, 26, 29]. These studies show an increased prevalence in obese children compared to overweight children. In the study by Caserta et al., odds ratios for IR were calculated for obese and overweight boys and girls comparing to their normal weight peers. The odds ratios of 9.1 (95% confidence interval 4.0–20.4) and 13.2 (4.7–36.9) were reported for obese boys and girls and lower odds ratios of 2.4 (1.2–4.9) and 6.0 (3.1–11.9) were reported for overweight boys and girls, respectively [20]. These results show that with normal weight increasing to obesity the prevalence of IR is rising.

A higher prevalence rate of IR has been observed in girls compared with boys in 7 out of 13 studies reporting sex specific prevalence rates (Figure 3(a)) [16, 18, 19, 27–29, 31]. This is in line with the prevalence of T2DM, of which IR is a precursor, as population-based studies on the prevalence of T2DM in children and adolescents also show higher prevalence rates in girls [33]. Hirschler et al. found no significant sex-related differences in IR. In their study, IR was associated with BMI and pubertal stage only, and not with gender. Their findings suggested that higher values in IR in girls compared to boys could be due to differences in pubertal development [34]. A study by Moran et al. measured IR using the euglycemic insulin clamp in children at all Tanner stages. At all Tanner stages, girls were more insulin resistant compared to boys. According to Moran et al., this difference in IR between boys and girls could partially be explained by higher levels of adipose tissue in girls compared to boys. However, in an obese subpopulation no difference in IR levels was observed between boys and girls [35]. It is known that pubertal development starts earlier in girls compared to boys (Tanner stage 2 at 11.4–11.9 years versus 11.9–12.3 years, resp.) [36]. Therefore, boys and girls between 10 and 14 years of age might be at another Tanner stage. Since IR is related to pubertal stage [34, 37], a comparison between pubertal girls and boys of the same age might result in a higher prevalence rate for IR in girls, because of a higher Tanner stage. The best comparison between boys and girls in pubertal age would be based on Tanner stages instead of age. Unfortunately, prevalence rates related to Tanner stages were not reported in any of the studies, so we were not able to check the effect of puberty on the prevalence of IR.

Our review has some limitations that should be addressed. At first, we could not compare results and pool the data of different studies, because of the heterogeneity in definition of IR in the presented studies. However, we were able to present an overview of the currently available population-based studies, showing higher prevalence rates in girls compared to boys, and in overweight and obese children compared to normal weight children. Another limitation is that all included studies were conducted in recent years. All studies were published between 2004 and 2014 and the data were collected between 2000 and 2011. However, in eight of eighteen studies, the exact period of data collection was not mentioned [14, 15, 18, 19, 26–28, 30]. Therefore, we could not evaluate whether the prevalence of IR is rising along with the increasing prevalence of obesity and T2DM. Finally, as already discussed above, the influence of Tanner stage on prevalence of IR could not be studied because of a lack of data.

## 5. Conclusion

In conclusion, the overall prevalence rates of IR in population-based studies of children and adolescents ranged between 3.1 and 44%, which could be partly explained by the use of different methods and cut-off values to determine IR. The prevalence rate of IR was up to 68.4% in obese boys. Girls seemed to have higher prevalence rates of IR than boys, which may however be related to their earlier pubertal development. Consensus on the definition for IR in children is needed to allow for comparisons between different studies, and to assess the value of IR as a screening measure for children and adolescents with an increased risk of cardiometabolic diseases.

## Supplementary Material

The supplemental table contains an overview of all studies included in the review. Data provided in this table are the study method, sample size, age of participants, ethnicity (if reported), criteria to determine IR and overall prevalence rates. If available, prevalence rates for subgroups based on weight category (normal weight, overweight and obesity) and gender were presented.

## Figures and Tables

**Table 1:** Search strategies.

Database	Search strategy
PubMed	(“Insulin Resistance”[Mesh] OR insulin resistan^*∗*^[tiab] OR insulin sensitivity[tiab] OR (resistan^*∗*^[tiab] AND insulin^*∗*^[tiab]) OR metabolic syndr^*∗*^[tiab]) AND (“Prevalence”[Mesh] OR prevalence^*∗*^[tiab] OR “Incidence”[Mesh] OR incidence^*∗*^[tiab]) AND (“Child”[Mesh:noexp] OR “Adolescent”[Mesh] OR “Puberty”[Mesh:noexp] OR “Minors”[Mesh] OR Pediatrics[MeSH:noexp] OR child[tiab] OR children[tiab] OR child care[tiab] OR childhood[tiab] OR child^*∗*^[tiab] OR childc^*∗*^[tiab] or childr^*∗*^[tiab] OR childh^*∗*^[tiab] OR adoles^*∗*^[tiab] OR boy[tiab] OR boys[tiab] OR boyhood[tiab] OR girl[tiab] OR girls[tiab] OR girlhood[tiab] OR junior^*∗*^[tiab] OR juvenile^*∗*^[tiab] OR kid[tiab] OR kids[tiab] OR minors^*∗*^[tiab] OR paediatr^*∗*^[tiab] OR pediatr^*∗*^[tiab] OR prepubert^*∗*^[tiab] OR pre-pubert^*∗*^[tiab] OR prepubesc^*∗*^[tiab] OR pubert^*∗*^[tiab] OR pubesc^*∗*^[tiab] OR school age^*∗*^[tiab] OR schoolchild^*∗*^[tiab] OR teen[tiab] OR teens[tiab] OR teenage^*∗*^[tiab] OR youngster^*∗*^[tiab] OR youth[tiab] OR youths^*∗*^ OR Primary school^*∗*^[tiab] OR Secondary school^*∗*^[tiab] OR Elementary school^*∗*^[tiab] OR High school^*∗*^[tiab] OR Highschool^*∗*^[tiab])

Embase	(prevalence/ or incidence/ or (prevalence^*∗*^ or incidence^*∗*^).ti,ab.) AND (insulin resistance/ or insulin sensitivity/ or metabolic syndrome X/ or (resistan^*∗*^ and insulin^*∗*^).ti,ab. or insulin sensitivity.ti,ab. or metabolic syndr^*∗*^.ti,ab.) AND (child/ or boy/ or girl/ or hospitalized child/ or school child/ or exp adolescent/ or adolescence/ or puberty/ or pediatrics/ or (child or children or child care or childhood or child^*∗*^ or childc^*∗*^ or childr^*∗*^ or childh^*∗*^ or adoles^*∗*^ or boy or boys or boyhood or girl or girls or girlhood or junior^*∗*^ or juvenile^*∗*^ or kid or kids or minors^*∗*^ or paediatr^*∗*^ or pediatr^*∗*^ or prepubert^*∗*^ or pre-pubert^*∗*^ or prepubesc^*∗*^ or pubert^*∗*^ or pubesc^*∗*^ or school age^*∗*^ or schoolchild^*∗*^ or teen or teens or teenage^*∗*^ or youngster^*∗*^ or youth).ti,ab. or youths^*∗*^.ti,ab. or Primary school^*∗*^.ti,ab. or Secondary school^*∗*^.ti,ab. or Elementary school^*∗*^.ti,ab. or High school^*∗*^.ti,ab. or Highschool^*∗*^.ti,ab.)

Cochrane	((prevalence^*∗*^ or incidence^*∗*^) and ((resistan^*∗*^ and insulin^*∗*^) or insulin sensitivity or metabolic syndr^*∗*^) and (child or children or child care or childhood or child^*∗*^ or childc^*∗*^ or childr^*∗*^ or childh^*∗*^ or adoles^*∗*^ or boy or boys or boyhood or girl or girls or girlhood or junior^*∗*^ or juvenile^*∗*^ or kid or kids or minors^*∗*^ or paediatr^*∗*^ or pediatr^*∗*^ or prepubert^*∗*^ or pre-pubert^*∗*^ or prepubesc^*∗*^ or pubert^*∗*^ or pubesc^*∗*^ or school age^*∗*^ or schoolchild^*∗*^ or teen or teens or teenage^*∗*^ or youngster^*∗*^ or youth or youths^*∗*^ or Primary school^*∗*^ or Secondary school^*∗*^ or Elementary school^*∗*^ or High school^*∗*^ or Highschool^*∗*^)).ti,ab.

**Table 2:** Methods used to calculate insulin resistance.

Method	Parameters	Formula	Cut-off values (range)	Studies using the method
HOMA-IR	FPG, FPI	(FPG (mmol/L) *∗* FPI (mU/L))/22.5	2.1–4.0	[14–27]
FPI	FPI	NA	9.85–23.7 *μ*U/mL	[17, 19, 20, 28–31]
QUICKI	FPG, FPI	1/[log⁡(FPI (mU/L)) + log⁡(FPG (mg/dL))]	0.33–0.35	[17, 23]
FGIR	FPG, FPI	(FPG [mg/dL]/FPI [mU/L])	7	[23]
HOMA2	FPG, FPI	Computer model: HOMA2-calculator: http://www.dtu.ox.ac.uk/homa	2	[28]
McAuley index	FPI, triglycerides	(2.63 − 0.28 ln⁡[FPI] − 0.31 ln⁡[fasting triglycerides])	6.3	[28]

FPG: fasted plasma glucose; FPI: fasted plasma insulin; FGIR: fasted glucose insulin ratio; HOMA-IR: homeostasis model assessment (for insulin resistance); QUICKI: Quantitative Insulin Sensitivity Check Index.
